# Patterns and outcomes of current antitumor therapy for high-grade neuroendocrine neoplasms: perspective of a tertiary referral center

**DOI:** 10.1007/s00432-025-06126-9

**Published:** 2025-02-19

**Authors:** Philipp Melhorn, Julia Spitzer, Thomas Adel, Ladislaia Wolff, Peter Mazal, Markus Raderer, Barbara Kiesewetter

**Affiliations:** 1 Department of Medicine I, Division of Oncology, Medical University of Vienna, Waehringer Guertel 18–20, 1090 Vienna, Austria; 2 Department of Pathology, Medical University of Vienna, Vienna, Austria

**Keywords:** Neuroendocrine tumors, Neuroendocrine carcinoma, Platinum-based chemotherapy, CAPTEM, Prognosis

## Abstract

**Purpose:**

Patients with metastatic high-grade neuroendocrine neoplasms (NEN) have an unfavorable prognosis. Treatment patterns and therapy outcome are scarcely evidenced, especially considering the WHO classification updates since 2017, and were thus investigated in this study.

**Methods:**

This retrospective single-center analysis evaluated patients with neuroendocrine tumors grade 3 (NET G3) or neuroendocrine carcinomas (NEC) treated at the Medical University of Vienna since 2010. The primary endpoints were progression-free survival (PFS) and overall survival (OS) following first-line treatment.

**Results:**

A total of 80 patients were included, 53 (66%) had NEC and 27 (34%) NET G3. Thirty patients had pancreatic NEN (38%), 29 gastrointestinal NEN (36%), 20 an unknown primary (25%), and one gall bladder NEC. All patients had metastatic disease, and all but four received systemic therapy. Platinum/etoposide was the most frequent palliative first-line treatment in NEC (41/47, 87%) and capecitabine/temozolomide (CAPTEM) in NET G3 (14/27, 52%). Overall, the median PFS and OS from first line start were 16.1 and 43.9 months for NET G3 and 6.1 and 12.7 months for NEC, respectively. Median PFS for platin/etoposide in NEC was 6.1 months (overall response rate [ORR] 56%) and for CAPTEM in NET G3 16.9 months (ORR 46%). Irrespective of the limited sample size (n = 4–11), second-line median PFS was short in NEC (FOLFIRI 2.8, FOLFOX 2.6, CAPTEM 5.4, other 2.6 months) and longer in NET G3 (8.2–11.1 months).

**Conclusions:**

The present data from a large European NET center show that multiple treatment strategies are used in NEN and highlight the varying outcomes between NET G3 and NEC.

## Introduction

Therapeutic paradigms for high-grade neuroendocrine neoplasms (NEN) have evolved considerably in recent years. Since neuroendocrine tumors grade 3 (NET G3) were separated from neuroendocrine carcinomas (NEC) as a unique group of high-grade NEN in the World Health Organization (WHO) classification (Rindi et al. 2022), more tailored therapeutic strategies have gained broad use. While the chemotherapy platinum/etoposide continues to be the standard for NEC in the first line, NET G3 seem to benefit more from different approaches, including capecitabine/temozolomide (CAPTEM), streptozotocin/5-fluorouracil (STZ/5-FU), sunitinib, everolimus, and peptide receptor radionuclide therapy (PRRT) (Pavel et al. 2020).

It is becoming increasingly apparent that NET G3 and NEC constitute different disease entities with distinct clinical characteristics and dissimilar genetic as well as epigenetic features (Mafficini and Scarpa 2019; Yachida et al. 2022). This is mirrored in the respective disease course and in expected treatment outcomes (Donadio et al. 2023; Sorbye et al. 2023). Apart from histologic differentiation, a key variable for classification and treatment decisions in neuroendocrine neoplasms is the proliferative activity measured by the Ki-67 index (Pavel et al. 2020). In that regard, the NORDIC NEC study was a landmark paper, demonstrating that patients suffering from a NEN with a Ki-67 < 55% are less likely to experience a response to platinum-based chemotherapy than those with Ki-67 index > 55% (Sorbye et al. 2013). This cut-off value also reflects the clinical threshold between NET G3 and NEC quite well, as the poorly differentiated NEC occur most frequently with a Ki-67 index of 50% and higher (Heetfeld et al. 2015).

NEN of extrapulmonary origin are rare malignancies (Dasari et al. 2018), hence evidence is still limited, and there is a lack of large prospective studies. Thus, little is known about the optimal management of high-grade NEN in terms of treatment sequencing. A recent registry study from Germany involving 445 patients aimed to characterize the different treatment strategies employed for high-grade NEN (Luecke et al. 2022). Platinum/etoposide and CAPTEM were frequently used in the first line, but there was a wide range of later-line therapies, including over 40 different protocols as second-line chemotherapy (Luecke et al. 2022).

With the present study we aimed to fill a gap in evidence concerning current treatment patterns and outcome of high-grade NEN. Over the last decade, numerous studies have provided comparative outcomes of NET G3 and NEC, including the prospective Nordic NEC 2 study recently presented as an abstract (Sorbye et al. 2024). However, beyond platinum-based chemotherapy, treatment sequencing is insufficiently characterized. Our goal was to share our experience as a European Neuroendocrine Tumor Society (ENETS) certified center treating high-grade NEN and to offer up-to-date treatment results of a single-center cohort. In addition, we include a detailed literature review that places our findings in the context of the available data.

## Methods

### Patients

This is a retrospective analysis based on electronic health records (EHR) data of 80 high-grade NEN patients treated at the Medical University of Vienna between January 2010 and December 2023. The inclusion criteria were histologically confirmed NET G3 or NEC, extrapulmonary origin, and metastatic disease. Patients with mixed neuroendocrine-non-neuroendocrine neoplasms (MiNEN) or NEN of the lung (small cell lung cancer or large cell NEC) were excluded. The histologic diagnosis was made or re-evaluated by a reference pathologist (PM). Patient data were collected from the EHR system of the Medical University of Vienna and stored in a database built up with FileMaker (Claris International Inc.). These data included baseline patient characteristics (age, sex, Eastern Cooperative Oncology Group [ECOG] status, and symptoms), disease information (primary tumor site, metastatic sites, functional status, histological parameters, and staging), and treatment outcome data (response, PFS, OS, and possibly cause of death). This study had received approval from the Ethics Committee of the Medical University of Vienna (EK No: 1616/2021).

### Endpoints

The primary endpoint of this analysis was to determine the PFS and OS following first-line treatment of patients with extrapulmonary NEN G3 in a real-world setting. Secondary and exploratory endpoints included descriptive analyses of the patient cohort and subgroup analyses with regards to PFS and OS. Disease response and progression were established based on routine radiological assessment (as documented in the medical records) and in unclear cases as routinely assessed by a reference radiologist within our multi-disciplinary tumor board. The intervals for response evaluation using computed tomography (CT), magnetic resonance imaging (MRI), and/or positron emission tomography (PET) were in accordance with current guidelines, i.e., approximately 3 months apart. The overall response rate was the percentage of patients with radiologically assessed complete response or partial response. The PFS was the interval from treatment start to disease progression or death, and patients were censored at the date of last patient contact if no progression event was recorded. OS was calculated from the treatment start date (and date of diagnosis, if so designated) to the date of death. For survival analysis, only palliative treatments were of interest, which were defined as therapies administered in a non-curative intent to treat metastatic disease. Only systemic anti-tumor treatments with palliative intent were counted as a separate line of therapy, while radiotherapy and locoregional therapies as well as maintenance therapies and add-on therapies were excluded. In case of tumor progression, patients were started on next-line treatment as recommended by the treating physician or the multi-disciplinary tumor board of our specialized NET center.

### Statistical analysis

To calculate statistical measures, test hypotheses, and create plots, the statistical programming language R version 4.3.2 with the packages tidyverse (Wickham et al. 2019), networkD3 (Allaire et al. 2017), ggsurvfit (Sjoberg et al. 2024), lubridate (Grolemund and Wickham 2011), gtsummary (Sjoberg et al. 2021), gt (Iannone et al. 2024), survival (Therneau 2024), and ggbeeswarm (Clarke et al. xxxx) was used. Descriptive statistics included frequencies and percentages (for categorical variables) and ranges, means, and medians (for quantitative variables). The Kaplan–Meier method was used to calculate the PFS and OS curves and the log-rank test to compare subgroups. The prognostic value of clinically relevant factors was assessed in univariate Cox regression analysis, and the most significant factors were further evaluated in multivariable analysis. Fisher’s exact test and Wilcoxon rank sum test were used as appropriate. A two-tailed p-value < 0.05 was regarded as statistically significant. Median follow-up was determined using the reverse Kaplan–Meier estimator (Schemper and Smith 1996).

## Results

### Baseline characteristics

**Fig. 1:**
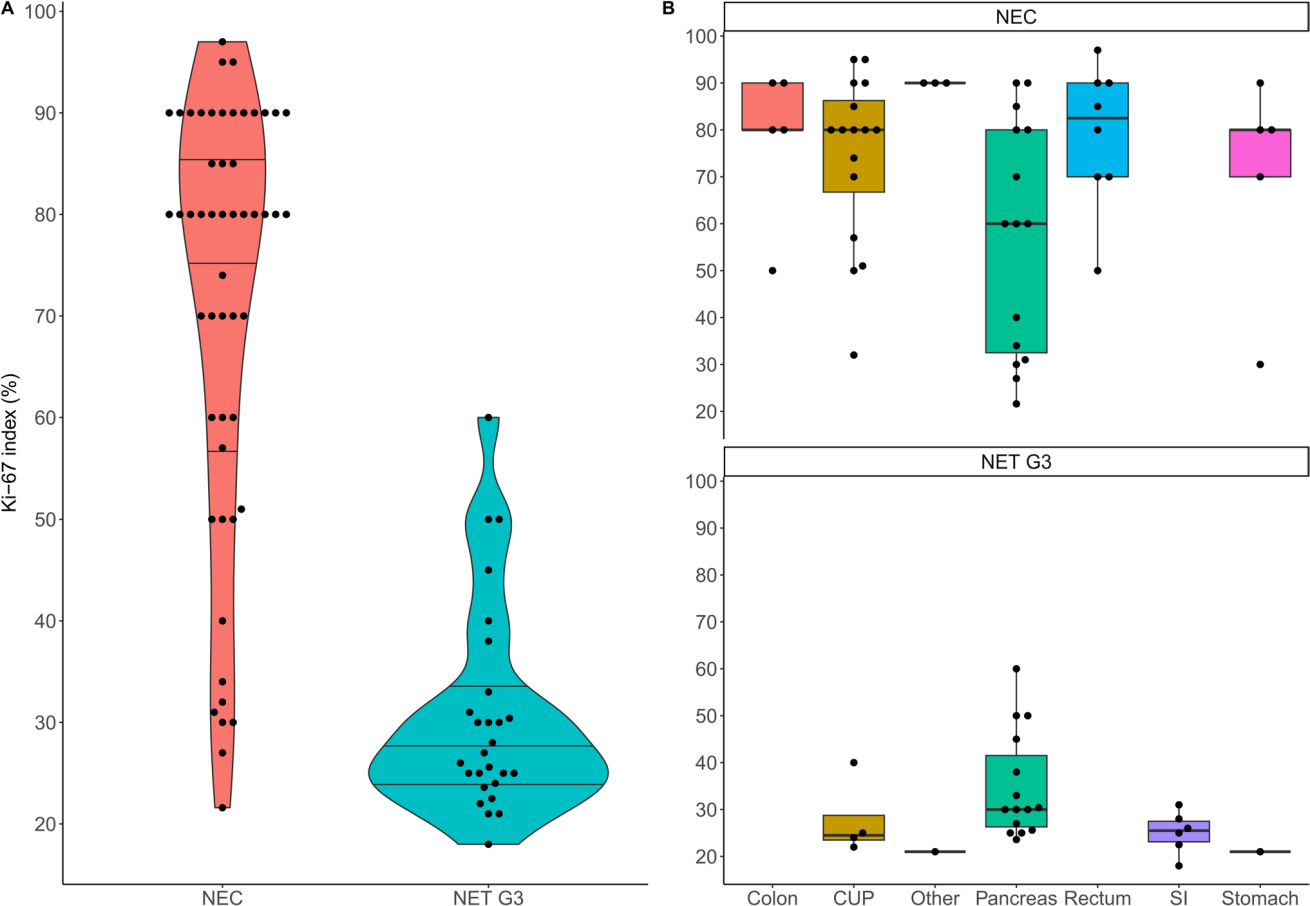
Ki-67 index (%) according to **A** histological subgroup (violin plot) and **B** primary tumor localization (box plots). The Ki-67 index was determined in 79/80 patients. One NEC patient had no Ki-67 index available, but a mitotic count of 20/2 mm^2^. One patient had a Ki-67 index of 18% and was diagnosed with NET G3 due to the high mitotic count (22 mitoses/2 mm^2^)

Out of 101 extrapulmonary high-grade NEN patients, 80 patients with metastatic disease could be included (pathological reassessment was not possible in seven, six had no metastases, five a MiNEN diagnosis, and basic information was missing in three). Of the included 80 patients, 42 (53%) were male. At diagnosis, the median age was 58 years (range: 25–80 years) and the ECOG performance status good in most patients (ECOG 0 in 88%). Twenty-seven patients (34%) had a diagnosis of NET G3 and 53 (66%) had NEC histology, of which 23 (43xxxx%) and 25 (47%) were of small cell (SCNEC) and large cell (LCNEC) morphology, respectively (not available [NA] for 5 patients). The median Ki-67 index of all patients was 60% (range: 18–97%) but differed significantly (Wilcoxon test: p < 0.001) between NET G3 (27%, range: 18–60%) and NEC (80%, range: 22–97%), with apparent differences between primary tumor sites, see Fig. 1. In total, 40/52 NEC patients (77%) had a high Ki-67 index (> 55%), while this was found in only one NET G3 patient (4%).

Stage IV disease was present at diagnosis in 66/80 patients (83%), 10 (13%) were in stage III, 3 (3.8%) in stage II, and 1 (1.3%) in stage I. All patients developed metastases during the course of the disease, with a median of 3 metastatic sites (mean number was 3.1 in NEC and 2.9 in NET G3), see Table 1. The liver was the most commonly affected organ (n = 68, 85%), followed by metastatic spread to the bone (n = 33, 41%) and lungs (n = 15, 19%). Seven patients had brain metastases (9%). Primary tumor site differed significantly between NET G3 and NEC, with cancer of unknown primary (CUP, n = 16, 30%) and pancreas (n = 15, 28%) being the most frequent in NEC and with pancreas (n = 15, 56%) as well as small intestine (n = 6, 22%) being the most common tumor origin in NET G3. Furthermore, 8 patients (10%) had a primary in the rectum, and 6 patients each (7.5%) in the colon and stomach. Four patients had CUP NET G3. One patient had a duodenal NET G3, one duodenal NEC, one esophageal NEC, and one patient gall bladder NEC, see Table 1.

**Table 1:** General patient characteristics

Variable	N	Overall, N = 80^*1*^	NEC, N = 53^*1*^	NET G3, N = 27^*1*^	p-value^*2*^
Sex	80				0.2
Female		38 (48%)	28 (53%)	10 (37%)	
Male		42 (53%)	25 (47%)	17 (63%)	
Age at diagnosis	80	58 (25, 80)	60 (26, 80)	55 (25, 73)	0.14
ECOG performance score	74				0.8
0		65 (88%)	39 (83%)	26 (96%)	
1		5 (6.8%)	4 (8.5%)	1 (3.7%)	
2		2 (2.7%)	2 (4.3%)	0 (0%)	
3		1 (1.4%)	1 (2.1%)	0 (0%)	
4		1 (1.4%)	1 (2.1%)	0 (0%)	
Histological subtype	53				
LCNEC			25 (47%)		
SCNEC			23 (43%)		
NA			5 (9.4%)		
Ki-67 index (%)	79	60 (18, 97)	80 (22, 97)	27 (18, 60)	** < 0.001**
Ki-67 index > 55%	79	41 (52%)	40 (77%)	1 (3.7%)	** < 0.001**
Disease stage	80				0.5
Stage I		1 (1.3%)	1 (1.9%)	0 (0%)	
Stage II		3 (3.8%)	1 (1.9%)	2 (7.4%)	
Stage III		10 (13%)	8 (15%)	2 (7.4%)	
Stage IV		66 (83%)	43 (81%)	23 (85%)	
Metastasis at any time point					
Distant metastatic disease	80	80 (100%)	53 (100%)	27 (100%)	
Number of metastatic sites		3 (1, 8)	3 (1, 8)	3 (1, 5)	0.8
Liver		68 (85%)	42 (79%)	26 (96%)	0.051
Bone		33 (41%)	21 (40%)	12 (44%)	0.8
Lung		15 (19%)	12 (23%)	3 (11%)	0.2
Brain		7 (8.8%)	7 (13%)	0 (0%)	0.089
Peritoneal carcinomatosis		10 (13%)	6 (11%)	4 (15%)	0.7
Other		14 (18%)	10 (19%)	4 (15%)	0.8
Distant lymph nodes		43 (54%)	29 (55%)	14 (52%)	0.8
Local lymph nodes		43 (54%)	30 (57%)	13 (48%)	0.5
Localization	80				** < 0.001**
Colon		6 (7.5%)	6 (11%)	0 (0%)	
CUP		20 (25%)	16 (30%)	4 (15%)	
Duodenum		2 (2.5%)	1 (1.9%)	1 (3.7%)	
Esophageal		1 (1.3%)	1 (1.9%)	0 (0%)	
Gall bladder		1 (1.3%)	1 (1.9%)	0 (0%)	
Pancreas		30 (38%)	15 (28%)	15 (56%)	
Rectum		8 (10%)	8 (15%)	0 (0%)	
Small intestine		6 (7.5%)	0 (0%)	6 (22%)	
Stomach		6 (7.5%)	5 (9.4%)	1 (3.7%)	
SSTR2-positive (IHC)	50	38 (76%)	19 (66%)	19 (90%)	0.051
SSTR-positive (imaging)	23	17 (74%)	4 (67%)	13 (76%)	0.6
FDG-positive (imaging)	20	17 (85%)	12 (92%)	5 (71%)	0.3
Functional symptoms	78	4 (5.1%)	0 (0%)	4 (15%)	**0.012**
Number of palliative systemic treatments	80	2 (0, 9)	1 (0, 4)	3 (1, 9)	**0.005**
Line of therapy (highest reached)	80				
0 (no palliative treatment)		6 (7.5%)	6 (11%)	0 (0%)	
1		29 (36%)	21 (40%)	8 (30%)	
2		17 (21%)	12 (23%)	5 (19%)	
3		17 (21%)	11 (21%)	6 (22%)	
4		5 (6.3%)	3 (5.7%)	2 (7.4%)	
5		5 (6.3%)	0 (0%)	5 (19%)	
9		1 (1.3%)	0 (0%)	1 (3.7%)	
Surgery	80	26 (33%)	15 (28%)	11 (41%)	0.3
Radiotherapy	80	15 (19%)	15 (28%)	0 (0%)	**0.002**
Liver-directed therapy	80	2 (2.5%)	1 (1.9%)	1 (3.7%)	> 0.9

^*1*^n (%); Median (Range)

^*2*^Fisher’s exact test; Wilcoxon rank sum test

Regarding the somatostatin receptor 2 (SSTR2) status, 90% of evaluated NET G3 patients were SSTR-positive (n = 19) on immunohistochemistry, whereas only 66% of NEC patients were immunohistochemically positive (n = 19). SSTR imaging by ^68^ Ga-DOTANOC PET/CT was performed in 23 patients (17 NET G3 and 6 NEC) at the time of diagnosis, with 13 (76%) of NET G3 and 4 (67%) of NEC patients showing a positive imaging result. ^18^F-FDG-PET was performed in 20 patients, and 5 (71%) of NET G3 and 12 (92%) of NEC patients had FDG-positive lesions. In total, 4 NET G3 patients (15%) exhibited hormone secretion (three had serotonin secretion with diarrhea or flushing and one had insulin hypersecretion), whereas no NEC patients analyzed showed hormonal symptoms (p = 0.012), see Table 1.

### Treatment patterns

All but 4 included NEC patients underwent systemic antitumor treatment (two with pending chemotherapy, one died before chemotherapy start, and one refused chemotherapy). Surgical primary tumor resection occurred in one third of patients (n = 26), and 15 NEC patients received radiotherapy. In total, 178 treatments were recorded. Two NEC patients had neoadjuvant cisplatin/etoposide, and 8 therapies were adjuvant (7 cisplatin/etoposide and one octreotide, three of them in NET G3). Of these 10 patients with neo-/adjuvant treatment, two NEC patients did not subsequently receive palliative therapy. In most cases, the time from diagnosis of metastatic disease to first-line palliative treatment initiation was short (median 1.2 months [NEC 0.9 and NET G3 1.3 months] and interquartile range 0.6–2.1 months [NEC 0.5–1.8 and NET G3 0.8–2.5 months]).

In the palliative setting, the first-line treatment in 47/74 patients (64%) was cis-/carboplatin in combination with etoposide, and it was the therapy of choice for most NEC patients (41/47, 87%) and about a quarter of NET G3 patients (6/27, 22%). The most frequently applied regimen in the first line for NET G3 patients was capecitabine/temozolomide (14/27, 52%), and it was also administered to four patients (8.5%) with NEC as an initial palliative therapy (3 had prior adjuvant platin-based chemotherapy). In line with good SSTR expression, SSTR-targeted treatment was given in 6 NET G3 patients initially (3 each had somatostatin analogs [SSA] or PRRT), and these therapies were also employed in later lines (second line: 1 PRRT, 2 SSA; third line: 4 PRRT, 3 SSA; fourth line: 4 PRRT and 2 SSA, fifth line: 1 PRRT), whereas PRRT and SSA were used only in individually selected NEC patients (5 PRRT across all therapy lines, including one PRRT + CAPTEM combination therapy).

**Fig. 2:**
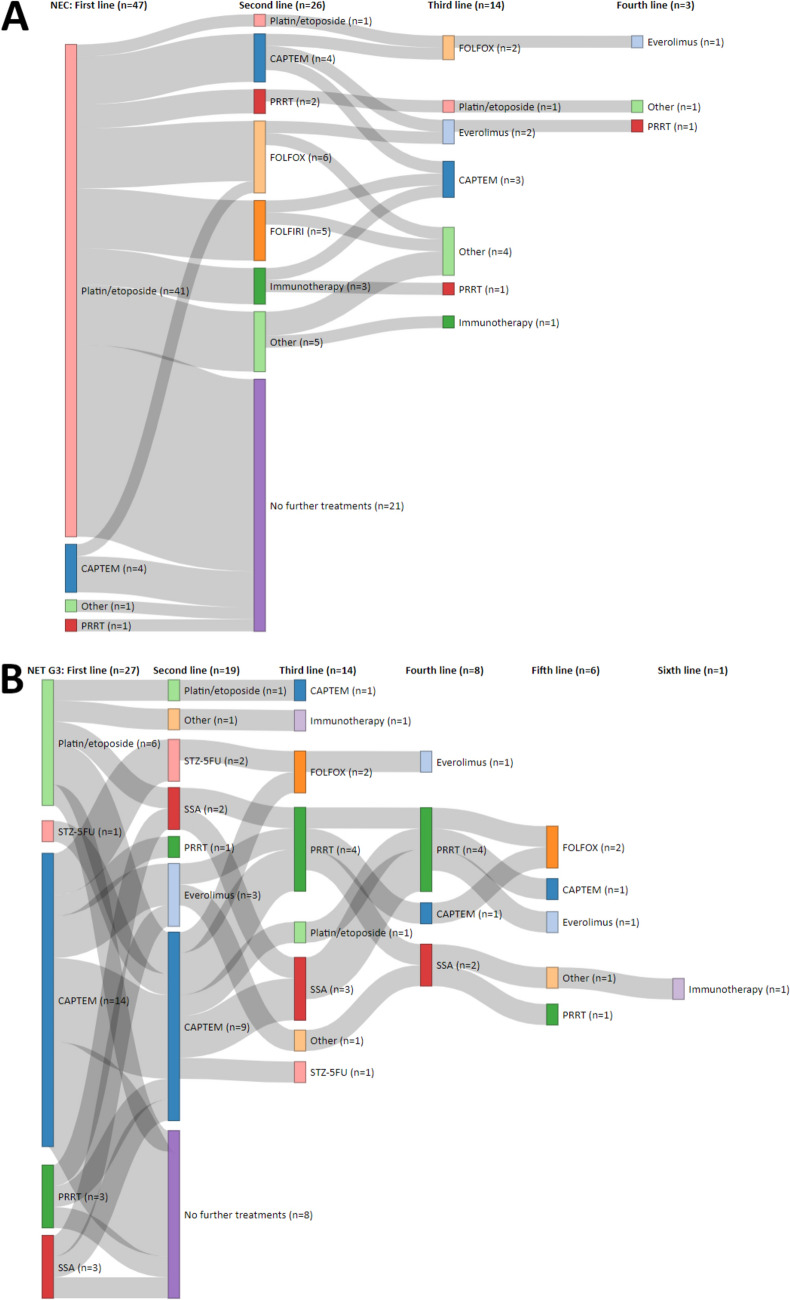
Sankey diagram of palliative treatment sequencing of patients with NEC (**A**) and NET G3 (**B**). CAPTEM, capecitabine/temozolomide. PRRT, peptide receptor radionuclide therapy. FOLFIRI, 5-fluorouracil/leucovorin/irinotecan. FOLFOX, 5-fluorouracil/leucovorin/oxaliplatin. STZ/5-FU, streptozotocin/5-fluorouracil. SSA, somatostatin analogs. Other administered therapies include FOLFOX + bevacizumab, STZ-doxorubicin, topotecan, sunitinib, ACO (adriamycin/cyclophosphamide/vincristine), capecitabine mono, EPICO (epirubicin, cyclophosphamide and vincristine), doxorubicin/5-FU, irinotecan mono, XELIRI (capecitabine/irinotecan), and cisplatin/temozolomide. Immunotherapies in use were nivolumab, pembrolizumab, and spartalizumab. One PRRT was administered in combination with CAPTEM

There was a significant difference in the number of administered palliative systemic treatments (median 1 for NEC and 3 for NET G3 and mean 1.7 in NEC and 2.9 in NET G3, Wilcoxon test: p = 0.005). Hence, NET G3 patients were much more likely to receive later lines of therapy, i.e., 14/27 NET G3 patients (52%) had a third palliative treatment, while only a third of NEC patients (14/47, 30%) reached such an advanced treatment line, see Fig. 2. Overall, 29/74 patients (39%) had no subsequent systemic antiproliferative treatment following first-line therapy (21 NEC patients and 8 NET G3 patients). Of those patients, 16 died during first-line treatment, 5 were lost to follow-up (last check-up more than 6 months ago), and 8 patients were still on first-line therapy at last visit. In total, 45 second-line treatments, 28 third-line, 11 forth-line, and 6 fifth-line therapies were recorded (one patient had 9 treatments), including 13 rechallenges (5 CAPTEM, 3 platinum/etoposide, and 3 Re-PRRT). With regards to the second line, CAPTEM was administered in 9 patients with NET G3 (following SSA, PRRT, platin/etoposide, and STZ/5-FU, and after CAPTEM as re-induction) as well as in 4 NEC patients upon disease progression following palliative platin/etoposide therapy. Platin/etoposide rechallenge was performed in one NEC patient. Twelve patients had second- or later-line treatment with 5-fluorouracil/leucovorin/oxaliplatin (FOLFOX) and five with 5-fluorouracil/leucovorin/irinotecan (FOLFIRI). For further information on the treatment patterns for this collective, including further chemotherapy regimens, everolimus, and immunotherapy, see Fig. 2.

### Clinical outcomes

The progression-free survival following first-line palliative treatment was 16.1 months in 27 NET G3 (95% CI 7.6–28.2 months) and 6.1 months (95% CI 5.3–7.6 months) in 47 NEC (p < 0.001), with no difference (p = 0.5 and p > 0.9 in the log-rank tests, respectively) between SCNEC and LCNEC (median 6.1 versus 4.8 months) and different primary tumor localizations (median for CUP 7.6, gastrointestinal 6.1, and pancreatic 6.2 months). There was a significant difference based on high versus low Ki-67 index (median PFS of 6.0 months versus 13.0 months, p < 0.001). The median OS following first-line palliative therapy was 18.7 months (95% CI 13.2–24.7 months), with a statistically significant difference (p < 0.001) between NET G3 (median OS of 43.9 months, 95% CI 24.7–NA months) and NEC (12.7 months, 95% CI 8.5–16.4 months) and between high (12.7 months, 95% CI 7.9–17.8 months) and low Ki-67 index (39.0 months, 95% CI 20.4–88.6 months). Regarding SSTR imaging in NET G3, there was a difference in PFS and OS (both p < 0.001) between SSTR-positive and SSTR-negative patients (n = 13 versus n = 4, median PFS 22.6 versus 3.1 months, and median OS 68.1 versus 19.6 months).

**Fig. 3:**
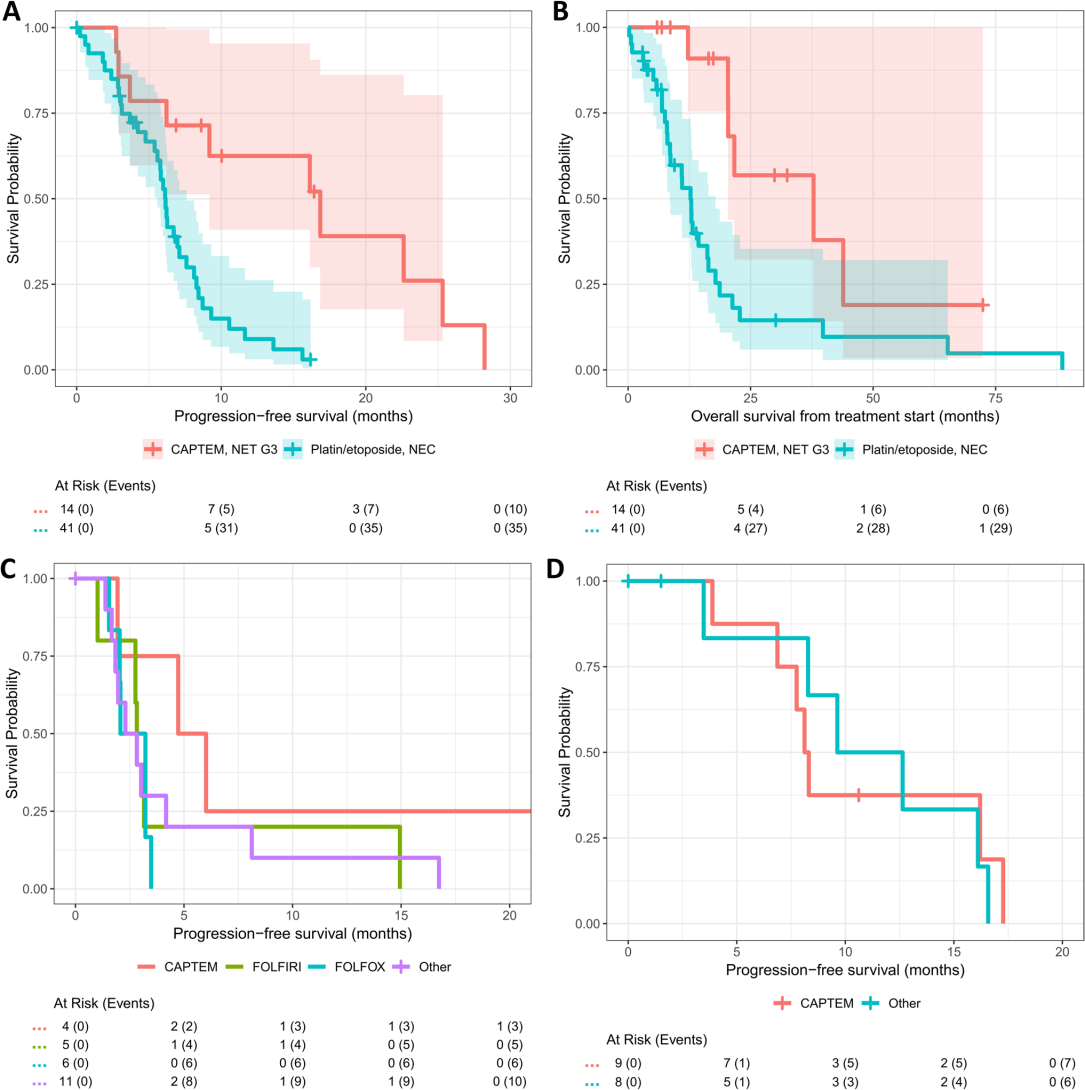
Kaplan–Meier analysis of PFS (**A**) and OS (**B**) from first-line palliative treatment start for CAPTEM in NET G3 and platin/etoposide in NEC as well as Kaplan–Meier analysis of second-line PFS for NEC (**C**) and NET G3 (**D**)

The median PFS with first-line CAPTEM in NET G3 was 16.9 months (95% CI 9.2–NA months) and the PFS with first-line platin/etoposide in NEC 6.1 months (95% CI 5.6–7.6 months), see Fig. 3A. The median OS from treatment start was 37.8 months (95% CI 20.4–NA months) for the former and 12.7 months (95% CI 8.5–17.8 months) for the latter, see Fig. 3B. There was no difference (p = 0.8) between cisplatin/etoposide (n = 32) and carboplatin/etoposide (n = 9), with a median PFS of 6.1 and 6.3 months, respectively. The overall response rate (ORR) with platin/etoposide in NEC was 56% (1/39 complete remission [CR], 21 partial remissions [PR], 8 stable disease [SD], and 9 progressive as best response, excluding 1 pending and one missing). By comparison, 6/13 NET G3 patients on CAPTEM had a partial remission (46%), 5 SD, and 2 only progressive disease (one missing). Most NEC patients (n = 14/39, 36%) had 6 cycles of platin/etoposide, with 7 patients given 8 cycles and 3 cycles applied in 6 patients (range: 1–9 cycles, 2 NA). There was no target number of CAPTEM cycles in NET G3 (median: 8.5, range: 2–36 cycles).

Factors associated with a worse first-line treatment PFS in univariate Cox regression analysis were male sex (HR 2.02), NEC histology (HR 3.31), high Ki-67 index > 55% (HR 2.94), ECOG performance status of 1 or worse (HR 2.70), and treatment with platin/etoposide (HR 2.98), while history of primary tumor resection (HR 0.42) and positive SSTR imaging (HR 0.21) were associated with better progression-free survival. In the multivariable analysis, male sex (HR 1.98) and surgery (HR 0.47) were independent prognostic factors for PFS. Regarding OS from first-line treatment start, NEC histology (HR 4.23), high Ki-67 index (HR 3.81), surgery (HR 0.50), ECOG ≥ 1 (HR 11.5), and positive SSTR imaging (HR 0.11) were significant prognosticators in univariate analysis, while NEC histology (HR 2.95) and ECOG ≥ 1 (HR 11.5) remained statistically significant in multivariable analysis, see Table 2.

**Table 2:** Cox regression analysis for PFS / OS following first-line treatment

Characteristic	Univariate (PFS)				Multivariable (PFS)			Univariate (OS)				Multivariable (OS)		
	N	HR^*1*^	95% CI^*1*^	p-value	HR^*1*^	95% CI^*1*^	p-value	N	HR^*1*^	95% CI^*1*^	p-value	HR^*1*^	95% CI^*1*^	p-value
Sex	74							74						
Female		–	–		–	–			–	–				
Male		2.02	1.17, 3.50	**0.012**	1.98	1.11, 3.53	**0.021**		1.13	0.63, 2.02	0.7			
Histology	74							74						
NET G3		–	–		–	–			–	–		–	–	
NEC		3.31	1.69, 6.50	** < 0.001**	1.50	0.38, 6.01	0.6		4.23	2.19, 8.15	** < 0.001**	2.95	1.11, 7.83	**0.030**
Ki-67 index > 55%	73							73						
No		–	–		–	–			–	–		–	–	
Yes		2.94	1.62, 5.35	** < 0.001**	2.07	0.91, 4.69	0.081		3.81	2.05, 7.07	** < 0.001**	1.33	0.54, 3.31	0.5
Primary tumor site	74							74						
CUP		–	–						–	–				
Gastrointestinal		1.04	0.52, 2.09	> 0.9					0.76	0.36, 1.62	0.5			
Pancreas		0.96	0.49, 1.86	0.9					0.51	0.24, 1.07	0.076			
Surgery	74							74						
No		–	–		–	–			–	–		–	–	
Yes		0.42	0.22, 0.79	**0.007**	0.47	0.23, 0.96	**0.038**		0.50	0.26, 0.97	**0.040**	0.58	0.28, 1.19	0.14
Functional symptoms	74							74						
No		–	–						–	–				
Yes		0.61	0.21, 1.72	0.3					0.61	0.22, 1.72	0.3			
ECOG at treatment start	66							66						
0		–	–						–	–		–	–	
1 +		2.70	1.01, 7.22	**0.048**					11.5	2.75, 48.2	** < 0.001**	11.5	2.68, 49.5	**0.001**
Positive IHC SSTR2	49							49						
No		–	–						–	–				
Yes		0.61	0.29, 1.28	0.2					0.56	0.25, 1.24	0.2			
Positive SSTR imaging	22							22						
No		–	–						–	–				
Yes		0.21	0.06, 0.72	**0.013**					0.11	0.02, 0.63	**0.013**			
First-line treatment	74							74						
CAPTEM		–	–		–	–			–	–				
Other		0.70	0.24, 2.02	0.5	1.42	0.44, 4.59	0.6		0.86	0.30, 2.47	0.8			
Platin/etoposide		2.98	1.48, 6.01	**0.002**	1.41	0.43, 4.62	0.6		2.00	0.95, 4.20	0.067			

^*1*^*HR* Hazard ratio, *CI* Confidence interval

Considering the limited sample size, progression-free survival intervals achieved with second-line treatments were short in NEC, with 2.8, 2.6, and 2.6 months for FOLFIRI, FOLFOX, and other treatments (including platin/etoposide and immunotherapy), respectively, whereas the 4 NEC patients on CAPTEM had a median PFS of 5.4 months, see Fig. 3C. In NET G3, CAPTEM (including 4 re-inductions) resulted in a median PFS of 8.2 months (other treatments 11.1 months), see Fig. 3D.

Across all treatment lines, the median PFS for therapies in NEC was in the range of 1.8–6.1 months, while in NET G3, therapies like everolimus, SSA, and PRRT led to median PFS intervals of 8.9, 12.1, and 15.7 months, respectively. Other chemotherapies (STZ/5-FU, platin/etoposide, and FOLFOX) only had median PFS durations of 3.0–3.6 months in NET G3. Table 3 shows the median PFS for different therapies across all treatment lines, but for some treatments only a few administrations were recorded.

**Table 3:** Progression-free survival across all palliative treatment lines

	NEC					NET G3				
	Records	Events	Median	0.95LCL	0.95UCL	Records	Events	Median	0.95LCL	0.95UCL
Platin/etoposide	41	35	6.1	5.6	7.6	7	5	3	2.1	
CAPTEM	10	8	5.3	4.3		23	18	14.1	8.3	22.6
Other	11	11	2.3	1.6		4	4	11.2	8.9	
PRRT	4	4	4.7	3.5		11	8	15.7	7.9	
FOLFOX	8	8	2.5	2		4	3	3.6	1.4	
Everolimus	3	3	3	1		4	2	8.9	1.7	
Immunotherapy	4	3	1.8	1		2	1	9.1		
FOLFIRI	5	5	2.8	2.8						
SSA						7	5	12.1	4.4	
STZ/5-FU						5	5	3.5	2.6	

PFS in months. Re-challenges have been excluded. LCL, lower confidence limit. UCL, upper confidence limit. NA were left bank

For the overall cohort, the median overall survival from diagnosis was 21.2 months (95% CI 15.2–39.7 months), and 53/80 (66%) patients had died at the data cut-off date after a median follow-up time of 63.8 months (95% CI 31.2–NA months) according to reverse Kaplan–Meier. In 48 of the deceased patients, the cause of death was attributed to tumor progression and consecutive organ failure, and this information was missing in five patients. There was a significant difference (p < 0.001) in OS between NEC (median of 13.4 months, 95% CI 10.8–19.4 months) and NET G3 (median of 44.7 months, 95% CI 39.2–NA months) as well as between high Ki-67 index (> 55%) with a median of 13.7 (95% CI 10.8–21.2 months) and patients with a Ki-67 proliferation index below that cut-off (median of 39.7 months, 95% CI 21.7–97.2 months). OS curves were similar between SCNEC (n = 23) and LCNEC (n = 25) with 13.7 versus 13.4 months median OS from diagnosis (p = 0.4).

## Discussion

This single-center analysis included a cohort of 80 patients with metastatic extrapulmonary NET G3 or NEC who were treated at a large European NET center. The main interest of this study was to evaluate treatment patterns and outcomes beyond the first line, especially considering the updated WHO classifications since 2017.

### Overall cohort

The primary endpoints were PFS and OS following palliative first-line treatment start, with the median PFS and OS being 6.1 and 12.7 months for NEC and 16.1 and 43.9 months for NET G3, respectively. The OS figures from our cohort are well in line with survival outcomes previously reported, i.e., about 33–44 months for NET G3 (Luecke et al. 2022; Elvebakken et al. 2021; Vélayoudom-Céphise et al. 2013; Hijioka et al. 2017; Milione et al. 2017; Zhang et al. 2017) and about one year for NEC (Elvebakken et al. 2021; Hijioka et al. 2017; Zhang et al. 2017; Taboada et al. 2022; Raj et al. 2017; Nielsen et al. 2020; Basturk et al. 2015; Shi et al. 2020; Tang et al. 2016). By comparison, Nordic NEC 2 (n = 698) found unexpectedly short OS durations after first-line chemotherapy of 7.4 and 21.8 months for NEC and NET G3, respectively (Sorbye et al. 2024). In contrast, the German NET Registry study by Luecke et al. (n = 445) reported OS from diagnosis (26 months in NEC and 44 months in NET G3 patients) for a cohort where 26.6% had locoregional disease (stages I-III) and 41.6% had surgery as the first treatment (Luecke et al. 2022). According to population-based data from the Surveillance, Epidemiology, and End Results (SEER) program, median OS was 33.9 for localized, 16.3 for regional, and only 5.2 months for distant gastrointestinal NEC (Dasari et al. 2018).

### Systemic treatment

A total of 168 palliative treatment lines were evaluated, with significantly more lines administered in NET G3 patients (mean of 2.9 versus 1.7 in NEC, p = 0.005). The choice of first-line palliative therapies in this collective resembles that of the treatment approaches in NEN G3 reported by the German NET Registry study (Luecke et al. 2022). Platinum-based chemotherapy was most frequently applied in NEC (72% versus 87% in our cohort) and administered to a third of NET G3 patients (33% versus 22%), while CAPTEM was used less frequently in NET G3 than in our cohort (32% versus 52%) (Luecke et al. 2022). As in this multicenter study, our results show that we did not use a universal first-line therapy for NET G3, but this was an individual decision. This reflects the wide range of possible treatment options recommended by current guidelines depending on tumor biology, SSTR status and tumor burden (Pavel et al. 2020).

In NEC, the median PFS with platin/etoposide was 6.1 months, the median OS 12.7 months, and the ORR 56%. This matches the median PFS of 5.6–6.4 months, the median OS of 11.3–12.5 months, and the ORR of 42–55% from two recent prospective evaluations of cisplatin/etoposide for NEC (Morizane et al. 2022; Zhang et al. 2020). For NET G3, data show superior PFS after capecitabine/temozolomide compared to NEC (median PFS 9.3 months versus 3.5 months, p = 0.005; median OS NA versus 6.2 months, p = 0.004; ORR 35% versus 14%, p = 0.393) (Jeong et al. 2021), and we observed a favorable median PFS of 16.9 months in our NET G3 subset (median OS 37.8 months, ORR 46%). For comparison, another retrospective analysis of 142 NET G3 patients found a median PFS of 6.9 months for both first-line platinum/etoposide and FOLFOX, while CAPTEM had a median PFS of 12.0 months (ORR 35% versus 56% versus 27%) (Apostolidis et al. 2021). The NORDIC NEC study found a lower ORR to predominantly platinum-based chemotherapy in patients with a Ki-67 index < 55% (15% versus 42%, p < 0.001) (Sorbye et al. 2013). Concordantly, Heetfeld et al. reported lower ORR in NET G3 (17%, 2/12 patients) than in NEC (35%, 39/113) (Heetfeld et al. 2015).

Our data reveal that—consistent with the absence of a clearly effective regimen in the literature—there is no clear favored treatment in the second line in our NEC patients, where the prognosis is often dismal (median PFS < 3 months and OS < 8 months according to one investigation) (McGarrah et al. 2020). In our analysis, CAPTEM seemed to achieve longer median PFS in NEC patients (5.4 months) compared to FOLFOX, FOLFIRI, and other treatments (< 3 months), however this finding has to be interpreted with caution due to the low number of patients and a potential selection bias. Regardless, this seems to support the use of CAPTEM in certain NEC patients, as per the relevant European guidelines (Pavel et al. 2020; Sorbye et al. 2023). On the other hand, FOLFIRI may currently have the best evidence with three recent phase II studies that failed to show superior results for the comparator arm: PRODIGE 41-BEVANEC (Walter et al. 2023) compared FOLFIRI ± bevacizumab, NET-02 (McNamara et al. 2023) studied Nal-IRI/5-FU/LV versus docetaxel, and SENECA (Bongiovanni et al. 2024) randomized patients to CAPTEM or FOLFIRI (again, median PFS < 4 months and median OS of < 9 months). Conversely, in our NET G3 patients, CAPTEM led to a longer median PFS in the second line (8.2 months) and performed similarly well in comparison to other second line treatments, also in view of the 4 CAPTEM re-inductions.

Unfortunately, and most likely explained by the biology of the disease, our patient numbers are relatively small for advanced treatment lines. Across all lines, the pooled PFS results for the different therapies suggest that selected NET G3 patients deemed to potentially benefit from SSTR-targeted treatment (PRRT or SSA) or everolimus have relatively long PFS intervals (median PFS of 15.7, 12.1, and 8.9 months, respectively). In comparison, NET G3 patients given chemotherapy other than CAPTEM (e.g., platin/etoposide, FOLFOX, and STZ/5-FU) had a disappointing PFS of often < 4 months—therefore the outcome may primarily reflect the expected disease dynamics. Results from previous studies concerning PRRT and chemotherapy in NET G3 may substantiate this conclusion (though data on SSA show shorter median PFS and evidence regarding everolimus is lacking) (Donadio et al. 2023). Based on our data, no statement can be made as to which therapy sequence is advantageous.

### Prognostic factors

Currently recognized prognostic factors for NEC include primary site, small-cell morphology, Ki-67 index, metastatic disease, elevated lactate dehydrogenase (LDH) and alkaline phosphatase (ALP) levels, and performance status (Sorbye et al. 2023; Lamarca et al. 2017). Separate evidence for NET G3 cohorts is limited: Apostolidis et al. (n = 142) identified only the use of platin/etoposide versus other treatments as a significant risk factor for progression (but not sex, primary site, SSTR positivity, LDH elevation, as well as other factors) (Apostolidis et al. 2021).

In our cohort, NEC histology and high Ki-67 index (> 55%) were significant prognostic factors for PFS (HR 3.31 and 2.94, respectively) and OS (HR 4.23 and 3.81, respectively), with histology being an independent prognosticator for OS in multivariable Cox regression analysis. The Ki-67 index cut-off value of > 55% was chosen based on previous studies such as the NORDIC NEC study (median OS of 14 versus 10 months, p < 0.001) (Sorbye et al. 2013) and Milione et al. (p < 0.0001) (Milione et al. 2017) and resulted in a clear separation of the survival curves of our patients (median OS of 43.9 versus 12.7 months, p < 0.001).

Furthermore, surgery and ECOG 0 indicated a favorable OS in our patient cohort, corroborating previous work by Elvebakken et al. who additionally reported pancreatic primary tumor, synchronous metastases, and normal ALP levels as prognostic for increased OS from metastases (Elvebakken et al. 2021). Similarly, positive SSTR imaging was significantly associated with better PFS and OS in the NET G3 subgroup, although this was available only for a small fraction of our patients (and not representative in the NEC subset). For comparison, in a larger study of high-grade NEN (n = 163), immunohistochemical SSTR-2a expression had no prognostic value overall but was associated with better OS in the pancreatic subgroup (14 versus 7 months, p = 0.02) (Nielsen et al. 2020). Finally, males had a higher risk of disease progression or death in our cohort. While this was not observed with regards to PFS in a previous NET G3 study (Apostolidis et al. 2021), male sex has been linked to inferior OS in multivariable Cox regression analysis in NEC (n = 2204) (Abdel-Rahman and Fazio 2021).

Neither PFS nor OS from diagnosis showed significant differences between SCNEC and LCNEC. Interestingly, Hijioka et al. reported a longer overall survival for SCNEC (11.3 versus 6.2 months, p = 0.036) (Hijioka et al. 2017) and NORDIC NEC found significantly longer PFS (5 versus 4 months, p = 0.021) but not OS (12 versus 11 months) for SCNEC (Sorbye et al. 2013). Contrarily, large-scale studies seem to indicate that small-cell morphology is associated with worse survival (Abdel-Rahman and Fazio 2021; Dasari et al. 2022).

Due to the retrospective design, this study is subject to all potential biases inherent in such analyses. First, these results may be limited by a selection bias, with patients treated at a tertiary referral center having baseline characteristics and outcomes that potentially differ from population-level data. Moreover, data quality could be impaired by incomplete or non-standardized health records. Unobserved confounding factor may have influenced the results. Furthermore, we have assembled a sizeable cohort of 80 NEN G3 patients with 168 palliative treatment lines recorded, however, this sample size largely precludes a reliable comparison of the individual treatment options. As a further limitation, the course of treatment represented the current real-world practice, with management on an individual basis for each patient and with some treatments initiated prior to initial presentation at our center.

In conclusion, there are several substantial differences between NET G3 and NEC, not only in clinical characteristics but also concerning treatment patterns and therapy outcome. It has been over half a decade since NET G3 was introduced into the classification of neuroendocrine neoplasms, and this real-world analysis shows that management of high-grade NEN according to morphology has been well established in clinical practice by now. Finally, the high number of treatment lines administered in our cohort suggests that these patients are best treated in an experienced center offering a wide range of treatment options.

## Data Availability

Enquiries for further data can be directed to the corresponding author.
